# The various aspects of genetic and epigenetic toxicology: testing methods and clinical applications

**DOI:** 10.1186/s12967-017-1218-4

**Published:** 2017-05-22

**Authors:** Ning Ren, Manar Atyah, Wan-Yong Chen, Chen-Hao Zhou

**Affiliations:** 1 Liver Cancer Institute and Zhongshan Hospital, Fudan University, Shanghai, 200032 People’s Republic of China; 2 Key Laboratory of Carcinogenesis and Cancer Invasion, Ministry of Education, Shanghai, 200032 People’s Republic of China

**Keywords:** Genetic toxicology, Genotoxicity, In-vivo testing, The Ames assay, Comet assay, Germ cells, Stem cells, Epigenetics

## Abstract

Genotoxicity refers to the ability of harmful substances to damage genetic information in cells. Being exposed to chemical and biological agents can result in genomic instabilities and/or epigenetic alterations, which translate into a variety of diseases, cancer included. This concise review discusses, from both a genetic and epigenetic point of view, the current detection methods of different agents’ genotoxicity, along with their basic and clinical relation to human cancer, chemotherapy, germ cells and stem cells.

## Background

Researchers have known that hazardous substances can interact with genetic materials, even before they have established a clear description of the genomic structure. Such substances can result in genomic instabilities and multiple mutations, which are associated with various kinds of diseases (including cancer). Those harmful substances include chemical, physical, and biological agents [1–3]. In genetics, genotoxicity refers to the ability of harmful substances to damage genetic information. It is often confused with mutagenicity, which refers to the permanent transmissible variations in the amount and structure of genetic materials of cells or organisms that can increase the frequency of mutations. Therefore, genotoxicity encompasses mutagenicity, but not all genotoxic substances are mutagenic, as they may not cause genetic alterations in DNA sequences.

Mutations are the permanent alterations in the DNA sequence of a cell’s genome and are caused by a battery of physical and environmental factors such as ionizing radiation, harmful viruses and hazardous chemicals [4, 5]. Moreover, errors during the DNA replication, repair, and recombination can also result in DNA mutations [6] like single point mutations and gene mutations (including base pair substitutions and add/del of a base), chromosomal aberrations (in structures and numbers), and genome mutations [7]. However, the exact molecular mechanisms of genotoxic substances that induce those mutations of genetic material are still unclear. Recent studies have shown that the genotoxic substances induce those damages or mutations through direct or indirect interactions with genetic materials [8–10]. It is widely accepted that several major DNA repair pathways [like direct repair, base excision repair (BER), nucleotide excisions repair (NER) and mismatch repair] are involved in the repairing process of hazardous substance-induced DNA damages leading to gene mutations [7]. For example, Benzene is a common environmental toxin, and its metabolite 1-4-benzoquinone (BQ) has been reported as a risk factor for hematopoietic cancers, such as myelodysplastic syndrome (MDS) and acute myeloid leukemia (AML) [11, 12]. To identify the most critical pathways that address BQ-induced DNA damage, a non-biased approach was performed in DNA repair-defective mouse embryonic stem cells by Son MY and colleague [13]. Consequently, they found that by directly suppressing type 1 topoisomerases, BQ inhabits replication fork restarts and progresses and, therefore, results in chromosomal instabilities which lead to hematopoietic cancers like MDS and AML. Other examples of genotoxic substances causing DNA damage are pyrrolizidine alkaloids (PAs), which are common constituents in plant species and are associated with diseases in human acquired from the consumption of contaminated food [14]. About half of them have been regarded as genotoxic. According to the results of genotoxicity testing, the researchers concluded that when metabolically activated, PAs produced a set of primary DNA and chromosomal damages. The major and signature types of mutations, this study pointed out, are G:C→T:A transversions and tandem base substitutions. Those results indicated that PAs are mutagenic and carcinogenic in vivo and in vitro, and their mutagenicity appears to be responsible for the carcinogenesis of PAs. In general, genotoxic substances may play an important role in carcinogenesis by directly and indirectly inducing many types of genetic damages.

To provide a technological and informational support for future researches, this concise review sheds some lights on the detection methods of the genotoxicity of different agents and its basic and clinical researches, based on the current literature and authors’ understanding.

## Testing techniques in genetic toxicology

**Fig. 1:**
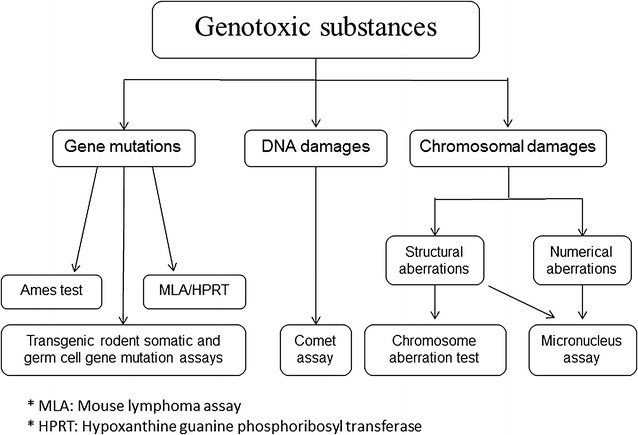
A battery of most common testing methods for the assessment of genotoxic substances

Genotoxicity assessment is an indispensable component in the safety assessment, aiming to prevent certain substances from affecting the human health. Since no single test is capable of detecting all relevant genotoxic end-points, a basic battery of in vivo and in vitro testing techniques for genotoxicity are recommended. Figure 1 shows some of the most common testing methods for the assessment of genotoxic substances. Historically, short-term tests (STTs) for evaluating the genotoxic potential of hazardous chemicals were introduced and modified decades ago. STTs include the Ames test [15], in vivo cytogenetics tests [16], and the micronucleus assays [17]. More recently, transgenic animal models have been established and proved to be powerful, organ-specific, short-term mutagenicity assays to explore the various steps involved in spontaneous or induced mutations [18, 19]. In addition, along with the rapid development of the next-generation sequencing technology, new methods have been introduced in genetic toxicology to directly analyze genetic materials in a genome-wide manner with single nucleotide resolution [20].

### In vivo testing

The purpose of in vivo testing is to determine the chemical’s potential DNA damage that can induce chromosomal loss or genetic damages. It can also detect a small number of genotoxic carcinogens which tested negative in in vitro tests. To date, a set of in vivo tests have been developed and widely used for genotoxicity, including in vivo comet assay, for detecting DNA damages [21]; in vivo micronucleus test, for chromosomal damage [22]; and transgenic mouse model assays, for mutagenicity [18]. Although the in vitro systems are more welcomed than the in vivo systems due to the growing concern on animal welfare, the in vivo test systems still need to be paid attention because of its weight of evidence.

### The Ames assay

The Ames assay, also known as the bacterial reverse mutation assay, is a rapid, highly-sensitive, and economic method for the detection of the mutagenicity of chemicals. As an alternative method to expensive and time-consuming animal tests, the Ames assay is developed in 1975 by Ames and his colleagues, and has been widely used in laboratories. This assay is performed on petri plates with several specially constructed strains of *Salmonella typhimurium* [15]. Those special strains are histidine-auxotrophic mutants (tester strains) that may hardly grow on a histidine-free medium, because they cannot synthesize histidine and must be provided by the surrounding. After adding mutagens, those mutants may revert to a “prototrophic” state (revertants), so that they can grow just fine on minimal agar plate. Thereafter, the mutagenic ability of the tested chemicals is assessed according to the number of revertants, which mainly depend on the number of colonies growing on the plate. The result of the assay detects a variety of genotoxic carcinogens, as well as different types of mutations such as frame shifts and base substitutions based on several tester strains [23]. Some researchers pointed out that little attention was given to dealing with positive results in the Ames assay, mainly due to the low specificity [24]. As a consequence, a positive result creates a significant obstacle in developments of new drugs. Although studies have shown that, compared to forward mutation assays, the major advantage of reversion assays is the well-defined nature of the mutagens; however, Ames assays come with two main disadvantages that hamper the expansion of this method: (1) the amount of the hereditary information in microorganism is less than that in mammals, and the structure of genetic material is more simple; (2) mammals have a more complicated DNA repairing system than microorganism.

### Comet assay and micronucleus assay

Among the several available detecting techniques for genotoxicity, comet assay (CA) and micronucleus assay (MNA) are two of the most common tests for genotoxicity, mainly due to their simplicity, sensitivity, and versatile trait to measure DNA strand breakage or chromosomal loss (which can be recognized as potential hallmarks of mutagenicity). Furthermore, recent studies have shown that a combined micronucleus and comet assay is the best technique in clarifying the mechanism of action of genotoxic compounds [25], since both assays have proved to be extremely sensitive and effective in detecting breaks at chromatic and chromosomal levels, respectively.

The comet assay, or single cell gel electrophoresis (SCGE) assay, was first introduced in 1984 as a micro-electrophoretic technique for the detection of radiation-induced DNA damage in individual mammalian cells. Briefly, a small number of radiated cells were embedded in a thin low melting point agarose gel on a microscope slide and lysed in a lysis buffer. The slide then was incubated in fresh alkaline buffer for a period of time. Subsequently, the DNA was electrophoresed and stained with a fluorescent DNA binding dye. Under the action of electric field, broken DNA fragments migrated from the nucleus and formed the pattern of migration, which often resembles a comet [26]. Finally, the fluorescence intensity and the length of the comet tail were measured to estimate the extent of DNA damage. According to different pH-values of electrophoretic buffer, Two variations of the comet assay exist: the neutral method (pH 8.4) and the alkaline method (pH > 13). The neutral method can only be used for the detection of double-stranded breaks (DSBs), and not for single-stranded breaks (SSBs). The alkaline method, on the other hand, can be used to identify both single and double stranded breaks, due to its higher sensitivity. However, some researchers have pointed out that the variability of the comet assay is a serious problem, and can affect its reproducibility [27, 28]. The most important reasons that influence the variability are the experimental conditions, including agarose concentration, electrophoresis time and voltage gradient. Other factors, such as lysis and alkaline incubation time, enzyme concentration and electrophoresis temperature, may also play an important role in the result. Therefore, greater attention should be paid to the conditions of the experience, not just the outcome.

The MNA, as one of the most successful and reliable tests in genetic toxicology, has been extensively used in toxicological screening for potential genotoxic compounds. More than a century ago, micronuclei (MN), also known as Howell–Jolly bodies, were introduced in cytoplasm of erythrocytes. Similar structures were described in the following studies and indicated that micronuclei can be recognized as markers for cytogenetic damages [29]. This significant finding suggested that micronuclei can be used as an assay system for the detection of chromosomal damages. Those studies elucidated that micronuclei is an important biomarker and the MNA is a reliable genotoxicity test for the detection of chromosomal loss or disruption of the mitotic apparatus. Compared to the CA, the MNA is faster, easier, and more reliable.

To date, the CA, as a soft and biological assay, has been used in many fields, such as: eco-genotoxicity [30], toxicology [31], pharmacology [32], and nutrigenomics [33]. Besides, the CA is also proposed to assess the levels of genetic damages in human diseases, like essential hypertension [34], chronic kidney disease (CKD) [35], type 2 diabetes [36], and even in cancer chemoprevention [37]. The MNA, on the other hand, has been widely used in molecular epidemiology, cytogenetic damages, and virology field. Recent studies pointed out that some viruses could increase the MN frequency through specific proteins. Cassel AP [38] attempted to investigate the relationship between HPV–DNA and the MN frequency in 158 normal cervical smears. He found that HPV is obviously correlated with higher levels of the MN frequency both in cytology and peripheral blood derived from infected women. Moreover, the MNA was also reported in the cancer research. Through evaluating the cytogenetic alterations in peripheral blood lymphocytes obtained from esophageal cancer patients, Emamgholizadeh [39] indicated that the MN frequency in patients treated with combined chemotherapy and radiotherapy was apparently higher than that in patients with radiotherapy alone, especially after 24 fractions of radiotherapy. Similar findings were also observed in other studies [40]. Those results show that the micronucleus assay is an effective tool for detecting chromosomal and cytogenetic damages and can be recommended as a routine clinical application for cancer patients.

Dose-selection and quantitative risk assessment are also very important when discussing genotoxicity testing. Therefore, groups like the International Workshop on Genotoxicity Testing emphasized on using all available data and appropriate mathematical models to characterize the dose–response relationship in quantitative risk assessment. The group concluded that a threshold does exist for exposure levels with no “direct DNA reactivity”. However, exposures with such reactivities can be of a certain risk at any given level [41, 42].

## Genetic toxicology in cancer cells and chemotherapy

According to what have already been mentioned, the assessment of the potential of genotoxicity is an essential step for a safe evaluation of hazardous substances. Various studies on the assessment of those substances have been performed in specific tissues or cells, such as liver cancer cell, lung cancer cell and prostate cancer cell [43–45]. For example, malathion is an organophosphate pesticide with high efficiency and low toxicity to humans and other mammals. Previous studies indicated that oxidative stress (OS) may be a possible mechanism of malathion toxicity in humans [46]. To investigate the definite role of OS in malathion-induced cytotoxicity and genotoxicity, a comet assay was performed by Moore [45] to evaluate the level of DNA damage in human liver carcinoma (HepG2) cells. They found that Malathion is mitogenic at lower levels of exposure, but also cytotoxic at higher levels.

With the development of cancer researches, anticancer drugs have been widely used for the treatment of various types of human malignancies [47, 48]. In general, a large number of patients with advanced cancer receive chemotherapy as part of their treatment. Genotoxic chemotherapy is the treatment of cancer by using one or more genotoxic drugs, which aims to induce DNA damage into cancer cells and thus kill those cells [49, 50]. However, It has been previously shown that most chemotherapeutic drugs are not specific against tumor cells and can also affect normal cells [51], which may lead to a wide range of severe adverse reactions [52, 53]. Therefore, while rapidly dividing cancer cells are extremely sensitive to drug therapy; normal functioning cells are also affected. Moreover, studies has concluded that many anticancer drugs such as alkylating agents [54, 55], topoisomerase II inhibitors [51], and thiopurines [56] can not only cause cell death, but also induce sub-lethal mutations in normal somatic cells, eventually giving rise to therapy-related secondary cancers [57]. For example, a study by Benjamini et al. showed that patients with chronic lymphocytic leukemia (CLL) who received frontline fludarabine, cyclophosphamide, and rituximab (FCR) therapy had 2.38 times higher risks of second cancers when compared to general population, with higher incidences of AML and MDS [57]. Therefore, it is necessary to assess the genotoxicity of anticancer drugs. Up to now, there are a variety of in vivo and in vitro genotoxic tests for monitoring the genotoxicity in patients receiving chemotherapy, such as the PIG-A assay [58].

## Genetic toxicology in germ cells

The invention and developments of current testing assays have led to a huge shift in the focus of genotoxicology from germ cells to somatic cells and their correlation with cancer, since it is easy to detect somatic cells’ mutagens with short term assays. In addition, the lack of sensitive and effective novel methods for detecting genotoxic mutagenic effects in germ cells made it harder to identify human cells’ mutagens (the majority of studies use model animals like rodents and cannot easily translate the novel findings into clinical use). Yet, more and more studies are trying to shed the light on the existence and importance of such mutagens, since a single mutation in a germ cell could potentially lead to a variety of diseases, while somatic cells mutations require a certain accumulation prior to any cancerous change [59, 60].

Therefore, efforts have been presented to determine whether somatic cell mutations and tests could be useful in predicting germ cells mutations. Although caution is required when determining such a relation, some suggest that somatic cells’ findings can indeed be useful to predict germ cells mutations; however, we should keep in mind that germ cells can have mechanistic and/or chemical-specific effects, and a potential sexual dimorphism may still exist. Thus, studies suggest that an exposure of germ cells or gonadal tissues to certain mutagens (even with negative somatic cells mutagenicity testing) can be a strong indication for germ cells mutagenicity testing; however, for somatic mutagens that do not reach gonadal tissues, such testing may not be necessary. When the somatic mutagens are believed to affect germ cells, testing are required for both establishing a diagnosis and quantitative risk assessments. Still we should keep in mind the downsides of repeated germ cell testing like germ cells loss, decreased sperm counts and reduced fertility [60].

All of that created a need for novel testing methods and assays that can detect germ cell’s DNA and chromosomal damages induced by mutagens (which can be transmitted to following generations), and pre-mutational changes (which have the potential to result in de novo mutations). That led to the invention of novel assays like: sperm and pedigree tandem repeat mutation analysis, high-throughput screening and DNA microarrays, in addition to novel endpoints added to researches like: chromosomal aneuploidies and structural aberrations, copy number variation (CNV) and single nucleotide variant (SNV), tandem repeat mutations, insertions/deletions, and mutations in non-coding sequences such as repetitive elements [60, 61]. However, those novel approaches also have limitations and are still under development. For example, the cost and the sample size required for CNV testing can still affect the spread and the use of this technology, although a great amount of genetic variations and mutations are believed to be related to CNV [62]. Yet these technologies not only helped to discover novel mutations and improve the selection of endpoints, but it also shifted the focus of researches from only concentrating on dominant mutations (usually associated with detectable phenotypes) to revealing more about recessive mutations [62] and, therefore, opening a window for early interventions prior to any phenotypical changes.

The current advanced technologies also allowed us to figure out more about the effects of certain substances and chemicals on so many levels that were not possible before. For example, a study that used Micronucleus assay and Fluorescence in situ hybridization analysis to evaluate the effects of styrene (a potential human carcinogen used in the production of plastics and polyester resins) on chromosomes and DNA damages showed a significant increase in primary DNA damage in styrene exposed workers when compared to healthy subjects. It also found an age-related decline in sperm DNA integrity and a significant correlation between the genotoxicity biomarkers detected in both somatic and germ cells; however, due to the limitations of this study, drawing conclusions based on those findings was not possible [63].

More studies have also started to point out that the differences in germ cells genotoxicity between different chemicals and mutagens may be due to the cell stage-dependent differences in DNA damaging and repairing. A study investigated the alkylation-induced germ cell mutagenesis in both mice and Drosophila found that for many species, an efficient DNA repair of pre-mutational damage is possible in early male germ cells and post-fertilization eggs; however, such a repair is not as effective in late spermatids and spermatozoa. In other words, a late-stage exposure of germ cells to certain mutagens may not be faced with any effective DNA repairing mechanisms and, therefore, could lead to higher rates of genotoxicity [64]. The study also found that for some mutagens (such as *N*-ethyl-*N*-nitrosourea), the effects are stronger in spermatogonial stages, while others (like methyl methane sulfonate) can be more mutagenic in post-meiotic stages. However, the type of DNA damage induced by the mutagen is also of a certain value when evaluating mutagenic effects of chemicals and not only the stage of interference alone. Retrospective analysis of chemical mutagenesis experiments found many more large deletions than other lesions in post-spermatogonial stages compared to spermatogonia; however, conclusions drawn from these studies should also be addressed with caution since limitations like inter-species comparisons and quantitative comparisons are present [64].

Another study also proposed synaptonemal complex damage as a measurement tool for genotoxicity at meiosis, presenting synaptonemal complex (SC) analysis as an approach for detecting meiotic chromosome anomalies and evaluating mutagenic mechanisms in chemical screening. The study found that chemicals (like the alkylators mitomycin C and cyclophosphamide) can affect late zygotene and pachytene cells leading to failed or irregular synapsis and breakage of whole SCs or their component axes. The effects of chemicals observed in this study were also stage-related, and a correlation between different types of SCs and metaphase chromosome abnormalities (e.g. deletions) were also found [65].

## Genetic toxicology in stem cells

Induced pluripotent stem cells (iPSCs) can be made by expressing a series of transcription factors in somatic cells which give back the pluripotency to these cells. They obtain many important characteristics (unlimited renewal and the ability to develop into different types of cells) that could translate into clinical advantages of less rejection rates in transplants and less ethical conflicts in some cases.

However, the chances of genomic alterations are present in many stages of the procedure of inducing pluripotent stem cells due to the epigenetic remodeling, aberrant expression of reprogramming factors, clonal selection, and prolonged in vitro culture. Therefore, nowadays monitoring the genomic stability of this type of cells is a main concern [66].

For the majority of chromosomally abnormal stem cells [embryonic stem cells (ESCs) in specific], apoptosis is the expected outcome. In addition, these cells are more sensitive to ultraviolet DNA damage and can initiate more effective DNA repairing mechanisms; therefore, they are considered to be more protected against genetic instabilities when compared to somatic cells. However, for a small number of cells, they can avoid apoptosis and maintain a pluripotent phenotype, regardless to the genetic abnormalities. Even more, some believe they may have more point mutations and karyotypical abnormalities than normally expected. Those genomic aberrations could happen in regions containing cell growth genes or survival related genes, which alter the behavior of affected cells on so many levels and increase the chances of abnormal cell growth and tumors [66]. Some studies also concentrated on comparing genetic instabilities in both ESCs and iPSCs, and linking certain abnormalities to these types of cells. A study found that chromosomal abnormalities like trisomy 8, 12 and 17 can be observed in stem cells, with trisomy 8 being more frequent in iPSCs and trisomy 17 only observed in ESCs [67]. Other studies found that the two types of cells obtain higher rates of CNV than somatic cells. They also succeeded in spotting some iPSC-specific CNVs and CNVs common in both types [68, 69]. However, those finding still cannot prove whether the reprogramming of stem cells is the reason behind the higher rates in genomic instabilities, since different studies conducted in different labs are still showing some inconsistencies in results [66].

The genetic abnormalities introduced above and other identified genome instabilities have limited the potential role of induced stem cells in clinical fields; therefore, many studies tried to identify the mechanisms behind those abnormalities, in order to provide more stable stem cells that can be safe enough for clinical use. Many theories have been proposed such us the selection of pre-existing mutations, reprogramming related and culturing related genotoxicity and vector related instabilities (including the architecture and the content of the vectors used) [66, 70, 71]. Of which, the vector related mutagenesis attracted a great attention and plenty of studies to research it. The use of vector proviruses is believed to be behind activating proto-oncogenes (in some cases) not only in the insertion sites but also in nearby areas (up to 50–100 Kb away) which can lead to phenotypical changes in target cell’s profile and result in premalignant and/or malignant cloning, and that is called “insertional activation”. However, some studies also believe that unless the insertion itself included a growth regulatory protein, only a small part of proto-oncogenes would potentially undergo such a transformation [70, 72]. For some cases (like lentiviral vectors), the interference could be in promoter and enhancer regions and, therefore, results in an inactivation of certain genes (like tumor suppressor genes) leading to a similar phenotypical outcome [72]. Up to this date, no vector is considered to be completely immune against insertional activation (or inactivation), and the safety of using certain vectors should be closely evaluated before any clinical procedure. However, the target cell characteristics also have an important role in the outcome. Yet, further investigation is strongly required to understand how those two links interact in the genetic alterations of both proto-oncogenes and tumor suppressor genes [72]. Besides, the needs of selecting the most fitted cells for culturing and the accelerated cell cycling could also compromise the DNA repairing and, therefore, increases the chances of genomic instabilities and abnormal growth of target cells [70]. Still, the clear identification of insertional sites of used vector plays a vital role in estimating and avoiding any potential malignant genomic alteration in cells. Therefore, many novel methods for tracing insertional sites of vectors have been invented since the first introduction of the complete human genome sequencing, such us: inverse polymerase chain reaction (IPCR), ligation-mediated PCR (LM-PCR), linear amplification-mediated PCR (LAM-PCR) and nonrestrictive linear-amplification-mediated PCR (nrLAM-PCR) [72].

Due to the great benefits iPSC can potentially bring to clinical therapies, more and more researches and efforts are being devoted to come up with methods and protocols to reduce the genotoxicity associated with iPSC. One of which is using clonally expanded cells in order to control the genetic integrity and limit the insertion-related mutagenic mutations, also trying to find the most suitable preclinical models to evaluate how safe and effective iPSC clinical application can be. However, the differences among species could make it hard to succeed in estimating the human immune reaction to a certain therapy approved in a different model. Yet, using nonhuman models in hematopoietic gene therapy did indeed help in the assessment of genotoxic events and made the clinical application of iPSCs in this field possible [66, 70].

Suggestions and efforts to standardize the genetic evaluation of iPSC have also been proposed, attempting to improve the current methods and to come up with novel methods that can deal with larger sizes of samples and with higher sensitivity to differences among iPSCs (since newly derived iPSC and genetically corrected iPSC clones may require different approaches when screening for abnormalities) [66]. Coming up with full classification of known functional mutations and suitable biomarkers and methods to detect them could also be so valuable to ensure a certain safety and effectiveness of iPSC clinical therapies, in addition to improving the quantitative analysis and quantitative assays for detecting mutations (since it is quite hard to completely avoid mutational abnormalities during stem cell’s inducing) [70]. Another important novel method is the “suicide gene strategy” which is considered as a safe exit when needed. By introducing a certain suicide gene to the induced stem cell, we can use it later as a “kill switch” when required; therefore, it provides more control of the induced cells, and an extra preventive measurement against any malignant behavior before or after transplantation [66, 72].

All of those methods mentioned above and many other methods related to cell’s culturing and cloning, vector’s modification and selection and other stages of inducing stem cells are still under development; however, with more and more studies and researches trying to improve the current techniques we are using, we may be close to finally applying the benefits of iPSC into clinical practice in a wider range and, therefore, improve the level of therapeutic services provided in many medical fields.

## Genetic toxicology and epigenetics

**Fig. 2:**
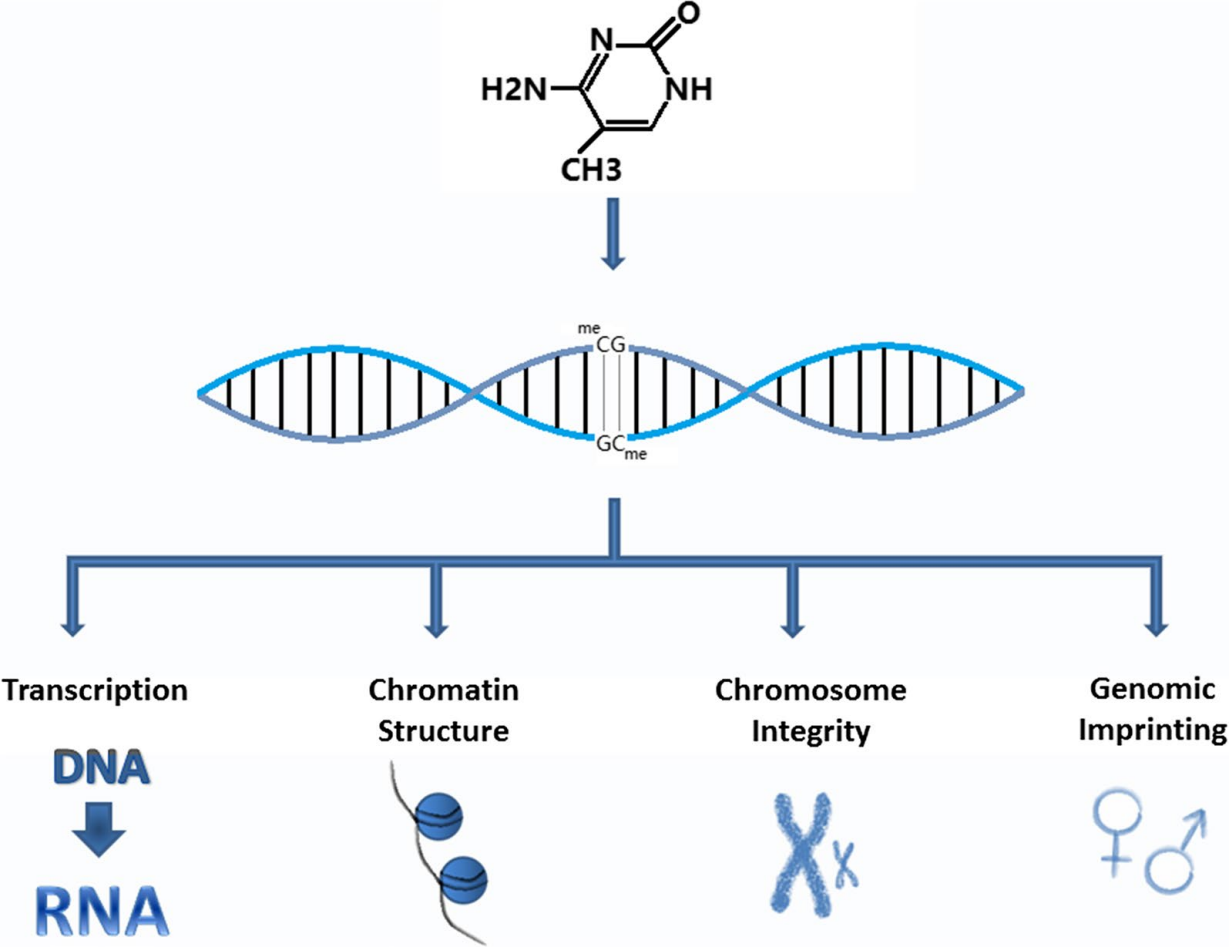
A diagram showing the structure and the effects of DNA methylation

Epigenetic alterations include: altered DNA methylation, histone modification, non-coding RNA and chromatin remodeling [73, 74]. More and more researches are pointing out the strong correlation between these alterations and human diseases like cancer, believing that epigenetic alteration stands for non-genotoxic mechanisms of carcinogenesis which may or may not be accompanied with genotoxic aberrations. Therefore, epigenetic Alterations have recently been added to the list of 10 “key characteristics of human carcinogens” [75]. Of these alterations, aberrant DNA methylation has been studied the most and is considered to be a common cause in many types of cancers. Figure 2 shows the molecular structure and the effects of DNA methylation. However, more studies are required to uncover more about the effects of epigenetic alterations on diseases’ phenotypes and dose–responses [73, 75].

The addition of methyl group to the 5th position of cytosine bases followed by a guanine bases (CpG) is called “DNA methylation”, it plays an important role in both regulating and determining transcription, chromatin structure, chromosome integrity and genomic imprinting. Therefore, any alteration of DNA methylation can interfere with these processes [75–77]. DNA methylation is mediated by three types of DNA methyltransferases (DNMT1, DNMT3A and DNMT3B). DNMT3A and DNMT3B are responsible for new methylation patterns in unmethylated DNA, while DNMT1 duplicate the methylation patterns in one strand methylated dsDNA during the DNA replication [75, 76]. Altered DNA methylation is considered to be a cancer hallmark and can be divided into two types: hyper-methylation and hypo-methylation. Hyper-methylation can happen in gene-specific promoters mostly in gene-rich genomic regions (CpG-islands) and lead to inhibition of gene expression (gene silencing), while hypo-methylation has been observed in repetitive elements all over the genome [75, 77].

Chromatins are made of dsDNA wrapped around core histones (primary structure), connected with histone free DNA. They then are folded into higher structures. Any changes in this “packaging” of chromatins can affect the accessibility of DNA to transcription factors and, Thus, interfere with gene expression and DNA repairing. That can happen by either changing the charge of histone proteins or by employing proteins that bind to specific modifications [75, 76, 78]. Several types of histone modification have been identified, such as: histone methylation, acetylation, phosphorylation, sumoylation, and ubiquitination of amino acids on the histone tails. Of which, histone methylation and acetylation have been studied widely [75, 79]. Histones methylation and acetylation is carried by special enzymes that can add or remove specific groups to histone proteins (called “writer and eraser” enzymes), therefore, changing the charge of histones and weakening the interaction with DNA, causing a relaxation of the chromatin that leads to transcriptional activation [like histone acetyltransferases, histone deacetylases (HDACs), histone methyltransferases and histone demethylases] [75, 76, 80].

It is well known that the greater part of human genome (approximately 60%) is transcribed into non-coding RNAs (NC-RNAs) which are not translated into protein. NC-RNA includes long non-coding RNAs (Lnc-RNAs) and microRNAs (miRNAs): two types that differ not only in length but also in functions. miRNAs are small, single stranded and act as translational repressors. It is estimated that about a third of the mammalian genes are regulated by miRNAs, which regulate gene expression by binding to the 3′ untranslated region of the gene and either induce RNA degradation or block translation of the gene. Such a regulatory action can be altered by many factors (like the exposure to environmental chemicals) and, therefore, play a vital role in different types of cancer [75, 76].

Based on the current knowledge regarding epigenetics, there have been many attempts to invest the novel findings and mechanisms in a wider clinical application, and that led to the invention of “epigenetic drugs” which are drugs that target certain epigenetic abnormalities on a cellular level. Many DNA methylation inhibitors have already been designed, some of which have been approved for clinical uses in diseases like myelodysplastic syndrome and hematologic malignancies; however, for the vast majority of these drugs, cytotoxicity and genotoxicity are unavoidable, with a potential of unwanted hypomethylation of oncogene promoters. Thus, a second-generation DNMTi are currently under development to be more sequence-specific and less toxic [78, 81]. Few histone deacetylase inhibitors (HDACi) have also been approved for the treatment of rare cutaneous T cell lymphoma and hematological malignancies. However, drugs of this category can also lead to DNA demethylation and not only interfere with histone modification [78]. Therefore, these drugs are in need of vital improvements regarding the safety of any clinical use. Other than “epi-drugs”, epigenetic has also been used in pharmacology to evaluate the cytotoxicity and genotoxicity of drugs during different preclinical development stages. Researches have been using endpoints like DNA methylation, histone modification degrees, and enzymatic activity of DNA methyltransferases to evaluate drug’s actions and efficacy [78].

Many methods have been developed to detect epigenetic alteration and mechanisms in genome wide and gene specific manners. Methods like: bisulphite conversion of DNA, methylated DNA immunoprecipitation, and chromatin immunoprecipitation (ChIP) have already been widely used. As for microRNA, microarrays or deep sequencing (for global expression), and real time PCR, northern blot or in situ hybridization (for specific miRNA identification) are suitable approaches [76].

Methylation assessment is considered to have a great value in epigenetic testing. It can provide us with more clear understanding of the toxicity of chemicals that cytolethality and genotoxicity considerations alone may not be able to provide. It is also useful in pharmacological researches where many medications fail to toxicity, causing a waste of time and resources; therefore, such an assessment may help prioritizing at early screening stages. Another advantage is that novel findings in methylation assessment researches can sometimes provide us with important clues regarding the development of anti-cancer agents, since we have a better understanding of the correlation between methylation and cancer than we used to have before. However, it is important to keep in mind that such an assessment is more valued when completed on a genome wide level and to be viewed as a component of an overall toxicity assessment, because alterations in DNA methylation may not necessarily be indicative of toxicity, in addition to the reversible nature of this process [82]. Methods like methylation-specific PCR (MSP), combined bisulfite restriction analysis (COBRA) for gene-specific DNA methylation, whole-genome bisulfite treatment with sequencing (WGBS), methylated DNA immunoprecipitation (MeDIP), and mass spectrometry for global levels of DNA methylation have already been used for the measurement of DNA methylation [75].

The recent improvements in this field have also covered the imaging technology. Novel methods like confocal laser scanning, two-photon excitation microscopy, high-content cell imaging, and digital tissue scanning have made “high-resolution optical imaging” an essential tool for testing new chemicals and made it possible to observe the distribution of molecules and cellular components within their native environment [78, 83].

**Fig. 3:**
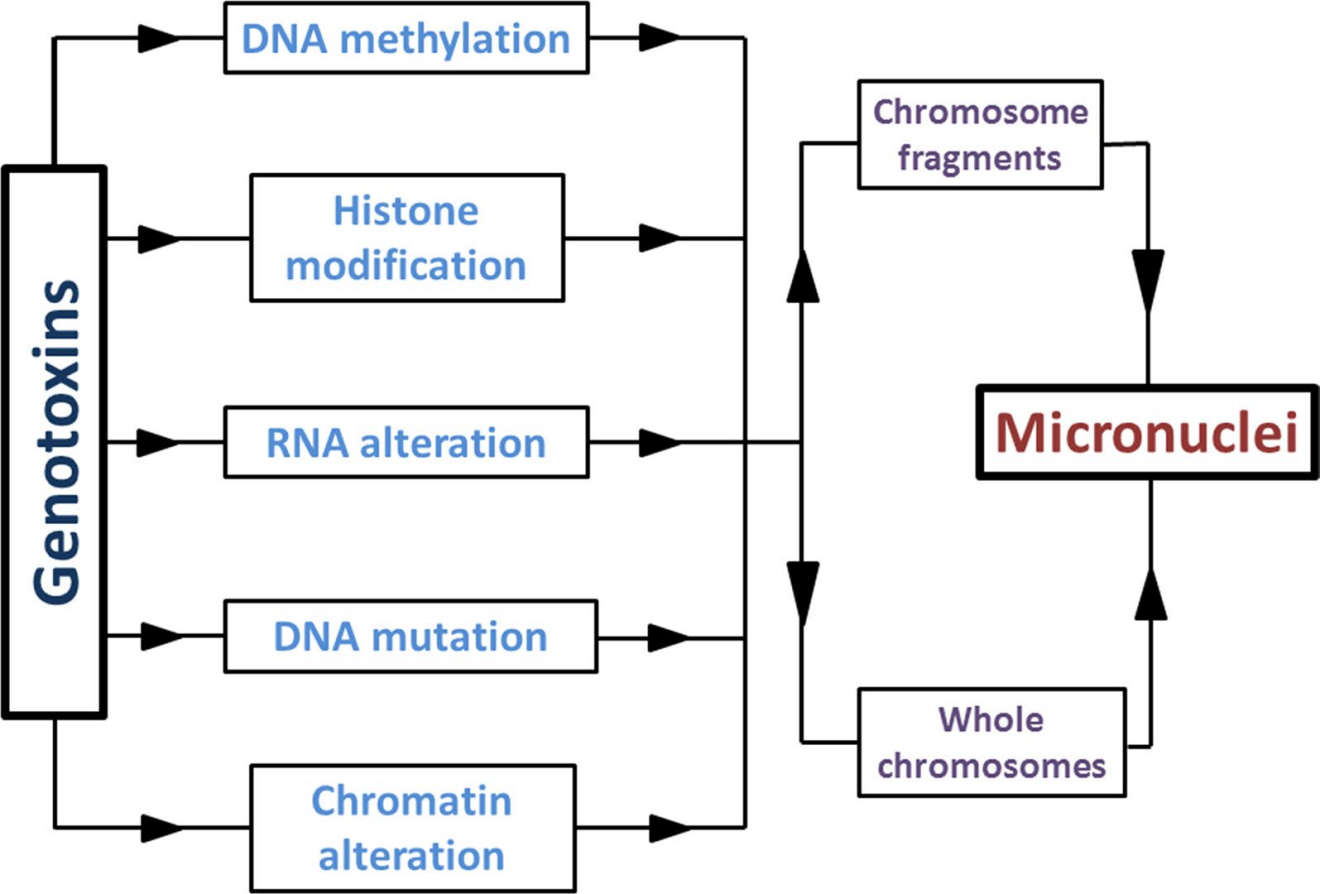
The contribution of different elements in forming micronuclei

3D quantitative DNA methylation imaging (3D-qDMI) is one of these novel methods that use fluorescence to visualize differential nuclear distribution patterns of methylcytosine and DNA, in order to detect drug-induced DNA demethylation and concurrent heterochromatin decondensation/reorganization in cells. This method introduces DNA methylation patterns as a potential pharmacodynamic biomarker of drug actions and, therefore, may be an encouragement for future assays that utilize chromatin structure genotoxicity [78]. Another novel approach in genotoxicity assessment is the detection of micronuclei (MN). Micronuclei (MN) (also known as Howell–Jolly bodies) are extra nuclear bodies which contain damaged chromosome fragments and/or whole chromosomes that were not incorporated into the nucleus after cell division. The presence of micronuclei can indicate accumulation in DNA damage and chromosomal abnormalities. Studies have indicated that the loss of DNA methylation could be a strong reason for the formation of micronuclei, in addition to histone modification and miRNA aberration. Figure 3 shows the contribution of different factors in forming micronuclei. However, more researches are required to clarify the exact contribution of each of these epigenetic mechanisms in the MN formation. This method has the advantages of easy detection and rapid formation of micronuclei. Besides, the content of micronuclei (chromosome fragments and or whole chromosomes) could shed some lights on the mechanism of action of mutagenic agents. However, this method is still under development, since the exact contribution of MN to gene expression and the mechanism of MN content degradation is still not clear. Yet, it has a great potential to improve the field of epigenetic testing and bring it another step forward [84].

A fair amount of recent studies have used the current improvements in epigenetic field to understand more about how a certain chemical or substance can interfere with human cells, especially for substances that are believed or suspected to obtain carcinogenic features. Here, we will shed some lights on few substances’ contribution in epigenetic alteration in order to understand epigenetic mechanisms in more detailed way.

Cigarette smoke components (like Nicotine, NNK, and PAHs) are well known for their cancerous effects, which can be induced by both genetic mechanisms (gene point mutation, deletions, insertions, recombinations, rearrangements, and chromosomal aberrations) and epigenetic mechanisms (alteration of cell proliferation and cell death functions). The Nicotine in cigarettes can behave like a cell growth factor and interact with special receptors [such as: nicotinic acetylcholine receptors (nAChR), *β*-adrenoceptors (*β*-AR) and epidermal growth factor receptor (EGFR)] and modify their levels of expression and sensitivity, leading to a hyper cellular response to growth factors than in normal cells [85]. Nicotine can interfere with important signal transducers that mediate signaling by many cytokines, growth factors, and onco-proteins [86]. It can also inhibit cells apoptosis [which is normally induced by tumor necrosis factor (TNF), ultraviolet (UV), radiation, or by chemotherapeutic drugs such as cisplatin, vinblastine, paclitaxel, and doxorubicin] by interfering with different pathways like PI3 K/AKT, Raf/MEKK/ERK1/2, and NF-κB, Bcl-2 [85]. Studies have also found that Nicotine can affect the angiogenic features of tumors by inducing the expression of growth factors (like VEGF, bFGF, PDGF, TGF-α, and TGF- β) which translates into migration and proliferation of tumor cells [85].

Benzo[*a*]pyrene (B*a*P) is another substance in cigarettes (in addition to several sources like automobile exhaust and heating with coal or wood) that is associated with lung, skin, bladder and esophageal cancers [75]. Studies showed that in some cases (B*a*P) is able to reduce the levels of global DNA methylation (in certain doses), while in other cases of tumor it is associated with hypomethylation of DNA repetitive elements. Hypermethylation of CpG islands within tumor suppressor genes has also been liked to (B*a*P) exposure. B*a*P also results in a global increase in acetylation of histones (like H3K9), and affects genes responsible for the organization and remodeling of chromatin. It also interferes with miRNAs involved in cell cycle arrest and the impairment of repair mechanisms of DNA damage. In a study using the human multiple myeloma cells, the exposure of (B*a*P) led to an up-regulation of 27 miRNAs, few of which are believed to have a role in repressing the p53 tumor suppressor gene [75, 87–89].

Nanomaterial (NM) has been defined by The European Commission as “a natural, incidental or manufactured material containing particles, in an unbound state or as an aggregate or as an agglomerate and where, for 50% or more of the particles in the number size distribution, one or more external dimensions is in the size range 1–100 nm” [76]. More than 1500 types of NM products are already in use in many fields (including health fields) regardless to the potential cytotoxicity and genotoxicity they acquire, which can interact (directly and indirectly) with lipids, protein and nucleic acids [76, 90]. Studies have found that NM exposure may lead to alterations in both global DNA methylation and specific gene promoters’ methylation levels, in addition to affecting enzymes that regulate DNA methylation (e.g. MBDs and DNMTs). Air pollution is also considered as a type of NM exposure. Studies regarding this matter found that mice exposed to air pollution had a 1.6-fold increased sperm mutations and DNA strand breaks compared to unexposed mice [91] in human observations, Hypomethylation of genes like *iNOS* and hypermethylation of *Foxp3* locus have been observed in adults and children exposed to air pollution [76, 92]. The ionic charge of NMs could also affect the charge of histone’s proteins which could alter the organization of chromatins, in addition to altering the function of histone modifying enzymes (e.g. HDAC) [76]. As for miRNA, NMs can alter its expression and functions and, therefore, affect genes involved in many vital cellular mechanisms (like miR-155 that has a role in regulating cellular pathways which are important in inflammation, carcinogenesis and cardiovascular diseases) [93]. However, in some cases, the effects are not immediate, a study by Chew et al. that used rats as a model observed miRNA changes 1 week and 2 months after blood exposure to gold nanoparticles (AuNPs). It found that 1 week after the exposure only 23 miRNAs were affected (like rno-miR-92b, and rno-miR-664), while 2 months after, the total number rose to 45 types (like rno-miR-214, rno-miR-327, rno-miR-466b, and rno-miR-494) [94].

As mentioned earlier, the exposure to certain substances can result in a variety of mutations and genotoxic abnormalities. Therefore, finding a certain testing method that can uncover all mutations caused by a given substance or compound is very challenging. Instead, combining an array of different methods, each directed to detect a certain mutation effectively, might result in a better outcome. These arrays can be designed according to already know or highly suspected mutations caused by the use of the substance itself or similar substances (whether they are drugs, chemicals, or nanoparticles).

## Conclusions

As mentioned earlier, genotoxicity is an aspect of certain substances that affect the integrity of both genetic and epigenetic information. Being exposed to many chemical and biological agents can result in genomic instabilities and/or epigenetic alterations, which translate into a variety of diseases, cancer included. Therefore, finding new effective testing methods to identify and measure the genotoxicity of given agents is quite important. However, to our knowledge, no single test is capable by itself of detecting all relevant genotoxic aspects and, therefore, a combination of different testing techniques should be used to achieve that goal. More researches are required to improve the current used methods, since many of which come with certain limitations, for example, the simplicity of genetic information and DNA repairing systems in microorganisms used in the Ames assay compared to mammals, and the variability of the comet assay due to different factors and conditions. The improvements in this field could also translate into clinical uses, considering how important testing methods and assays can be in monitoring the genotoxicity in patients receiving chemotherapy, or detecting adverse effects of certain drugs. More researches should also help improving our current knowledge in less explored areas of this field, like germ cell genetic toxicology. They should use the available data for new purposes in order to come up with new approaches and effectively include our findings in clinical use.

The epigenetic aspects of agents’ genotoxicity are also important to form a better understanding of how and when those agents affect the genome integrity; therefore, more epigenetic endpoints are being used in the assessment of diseases (like cancer). However, our current knowledge of which epigenetic alterations are most informative of specific damages or diseases and how to compare novel epigenetic markers to currently used markers is still limited. In addition, the plasticity of the human epigenetics can make answering those questions even harder [75].

Many limitations are restricting the role of the novel findings and approaches in this field. For example, the cost and the sample size required for some assays can prevent us from using them more widely. In addition, limitations of current studies like inter-species comparisons and quantitative comparisons in model animals and rodents’ studies could make it hard to draw conclusions based on the findings. Still, such studies are very important due to their potential in uncovering new aspects and mechanisms related to genetic toxicology, which can translate into wider more effective clinical use in many diseases, including cancer.

Therefore, it is safe to say that increasing the efforts and resources invested in researching genotoxicity from both genetic and epigenetic point of view would absolutely help us to step up the level and the quality of the health care provided in many aspects, considering the great potential of such researches in improving the diagnosis and the treatment of many current diseases and medical dilemmas.

## Abbreviations

AML: acute myeloid leukemia
BER: base excision repair
CA: comet assay
ChIP: chromatin immunoprecipitation
CKD: chronic kidney disease
DNMTs: DNA methyltransferases
DSBs: double-stranded breaks
EGFR: epidermal growth factor receptor
ESCs: embryonic stem cells
HDACi: histone deacetylase inhibitors
HDACs: histone deacetylases
HPRT: hypoxanthine phosphoribosyl transferase
IPCR: inverse polymerase chain reaction
IPSCs: induced pluripotent stem cells
LAM-PCR: linear amplification-mediated PCR
LM-PCR: ligation-mediated PCR
Lnc-RNAs: long non-coding RNAs
MDS: myelodysplastic syndrome
MeDIP: methylated DNA immunoprecipitation
miRNA: microRNAs
MLA: mouse lymphoma assay
MN: micronuclei
MNA: micronucleus assay
MSP: methylation-specific PCR
NC-RNAs: non-coding RNAs
NER: nucleotide excisions repair
NM: nanomaterial
OS: oxidative stress
SC: synaptonemal complex
SCGE: single cell gel electrophoresis
SNV: single nucleotide variant
SSBs: single-stranded breaks
STTs: short-term tests
TNF: tumor necrosis factor
